# Effectiveness of Lifelong ART (Option B+) in the Prevention of Mother-to-Child Transmission of HIV Programme in Zambia: Observations Based on Routinely Collected Health Data

**DOI:** 10.3389/fpubh.2019.00401

**Published:** 2020-01-17

**Authors:** Brian Muyunda, Patrick Musonda, Paul Mee, Jim Todd, Charles Michelo

**Affiliations:** ^1^Department of Epidemiology and Biostatistics, The University of Zambia School of Public Health, Lusaka, Zambia; ^2^Ministry of Health, University Teaching Hospital, Lusaka, Zambia; ^3^Faculty of Epidemiology and Population Health, London School of Hygiene and Tropical Medicine, London, United Kingdom

**Keywords:** PMTCT, pregnant women, option B+, routine data, HEI, Zambia

## Abstract

**Background:** Mother to child transmission of HIV (MTCT) is a global challenge affecting many countries especially in sub-Saharan Africa. In 2009 about 370,000 infants were infected with HIV mainly through MTCT and most of them in sub-Saharan Africa. We aimed to determine the effectiveness of Option B+ compared to other options in reducing rates of early MTCT of HIV infections in Zambia.

**Methods:** This was a retrospective cohort study based on routinely collected data using SmartCare in Zambia. Survival analysis with Cox Proportional Hazard regression was used to determine association between MTCT and regimen type of mothers. Kaplan-Meier (K-M) curves were used to compare MTCT for infants born to mothers option B+ to those on other options, and Wilcoxon (Breslow) test was used to establish statistical significance.

**Results:** Overall (*n* = 1,444), mother-baby pairs with complete data were included in the analysis, with the median age of mothers being 33 (28–38) years; and 57% of these women were on Option B+. MTCT rate was estimated at 5% (73/1,444) [*P* = 0.025]. A Kaplan-Meier estimate showed that HIV Exposed Infants (HEI) of mothers on Option B+ had lower MTCT rate than those who were on other MTCT prevention interventions [Wilcoxon test; chi2 = 4.97; *P* = 0.025]. Furthermore, The Nelson Aalen cumulative hazard estimates indicated similar evidence of option B+ being more effective than other options with some statistical significance [HR = 0.63, *P* = 0.068]. HEI of option B+ mothers had 50% reduced risk of having HIV infection compared to option A/B [adjusted HR = 0.4; 95% CI = 0.28–0.84; *P* = 0.010]. HEI to women who were married had an increased risk 50% of getting infected compared to those not married [adjusted HR = 1.5; 95% CI = 3.43–6.30; *P* < 0.001]. Exposed infants whose mothers had assisted delivery had 3 times increased risk of getting infected compared to those born through normal vaginal delivery [Adjusted HR = 3.2; 95% CI = 0.98–10.21; *P* = 0.050].

**Conclusions:** The use of Option B+ as PMTCT intervention was found to be more effective in reducing MTCT of HIV compared to other options. Scaling up access to life-long ART and improving retention for women on treatment can potentially reduce further vertical transmission.

## Introduction

Mother to child transmission of HIV (MTCT) is a global challenge affecting many countries especially in Sub-Saharan Africa. In 2009 about 370,000 infants were infected with HIV mainly through MTCT with most of them in Sub-Saharan Africa (1). By 2012, the number of newly infected infants globally had come down to 260,000 (2, 3) and by 2015, only 150,000 children were newly infected with HIV at birth [(4); UNGASS]. In Zambia, MTCT is one of the key drivers of HIV epidemic with 10% of all new HIV infections, and 90% of infections in children attributable to MTCT. Without antiretroviral therapy, 15–30% of babies born to HIV positive women are infected during pregnancy and delivery, while a further 5–20% become infected through breastfeeding (4–6). In resource constrained countries, approximately one third of HIV infected children die before 1 year and more than half die before their second year (7–12).

In 2008, 16.4% of women attending antenatal clinic (ANC) in Zambia were HIV positive, putting 80,000 infants at risk of getting infected through MTCT (5, 13). The Zambia ministry of health integrated PMTCT into Maternal and child health (MCH) to help reduce MTCT of HIV and to decrease both maternal and child mortality (4, 6). In an effort to further reduce MTCT of HIV, in 2013 Zambia adopted the World Health Organization (WHO) guidelines and introduced Option B+ as a new strategy within the PMTCT program. In the same year, the national PMTCT program recommended that all infants born to HIV positive mothers had a virological antigen test for HIV within the first 6 weeks and a second test at 6 months of life. HIV rapid antibody tests would only be used at the age of 12 and 18 months to check on the HIV status of the infants (14). Option B+ requires initiation of all HIV positive pregnant and breastfeeding women onto lifelong Antiretroviral therapy (ART), regardless of CD4^+^ cell count or WHO clinical staging.

Before adopting option B+, the Zambian National Guidelines for PMTCT, updated in 2007–2009, demanded that women eligible for lifelong combination Antiretroviral Therapy (cART), option A/B, were those with absolute CD4 count ≤ 350 cells/mm^3^ (regardless of clinical stage). Option A regimen included AZT starting at 14 weeks gestation followed by single dose Nevirapine (sd-NVP) and AZT/3TC at delivery for 7 days postpartum for mother and daily NVP from birth until 1 week after breastfeeding cessation or 4–6 weeks if no breastfeeding or mother on triple ART for the infant. On the other hand, Option B included Triple ARV Prophylaxis at 14 weeks gestation and ending at delivery or 1 week after breastfeeding cessation and Daily NVP or twice daily AZT for 4–6 weeks when replacement feeding and daily NVP for 6 weeks when breast feeding. This criteria had a negative impact on the effectiveness of option A/B regimen because of resource constraint challenges that included (1) capacity of health centers to assess CD4 count, (2) availability of CD4 count results at clinics for decision making, and (3) capacity to initiate cART. One study conducted in Zambia showed that test results of 33.5% of blood samples collected for CD4 count were never returned to the clinic. Only a minority of HIV-positive pregnant women were assessed for CD4 count and had their test results available. Among HIV-positive women whose CD4 count results were available, 47% were eligible for cART due to the cell count threshold of ≤ 350 cells/mm^3^. Frequent breakdown of CD4 count machines, insufficient number of trained laboratory technicians to run CD4 count laboratory equipment, lab fees applied in some facilities for CD4 count, and clerical errors all compounded the problem (4, 5, 15, 16). Women who were not eligible for lifelong ART were given a short course of prophylactic treatment designed to protect the infant from MTCT of HIV (Table 1).

**Table 1:** Treatment algorithm and transition of PMTCT strategies in Zambia for HIV positive women and their exposed babies.

	**Woman**		**Infant**	
	**CD4^**+**^ Cell count <350 cells/μl WHO staging 3 or 4**	**CD4^**+**^ Cell count ≥ 350 cells/μl WHO staging 1 or 2**		
Option A	ART for Life	AZT starting at 14 weeks gestation	Daily NVP from birth until 1 week after breastfeeding cessation or 4-6 weeks if no breastfeeding or mother on triple ART	
		sd-NVP and AZT/3TC at delivery for 7 days postpartum		
Option B	ART for Life	Triple ARV Prophylaxis at 14 weeks gestation and ending at delivery or 1 week after breastfeeding cessation	Daily NVP or twice daily AZT for 4–6 weeks when replacement feeding. Daily NVP for 6 weeks when breast feeding	
Option B+	ART for Life	ART for Life	Daily NVP for 6 weeks	

Option B+ initiative started in Malawi in 2011 because of the country's high HIV prevalence; short birth intervals (median = 3 years), high fertility (total fertility rate = 5.7), extended breastfeeding and a limited laboratory capacity (14, 17, 18). Zambia shared many of Malawi's characteristics for MTCT of HIV. However, there has been great controversy on the adoption of the option B+ strategy as the best approach to achieving elimination of mother-to-child-transmission from its inception in 2011 in Malawi and its adoption in other resource constraint regions with limited laboratory capacity. This study aimed to establish the effectiveness of option B+ compared to other PMTCT interventions and the factors associated with MTCT. The results will help fill the increasing gap between established policy on PMTCT strategies, particularly option B+ as an effective approach to reduce HIV transmission and the social practices associated with program feasibility, accessibility, uptake, and retention in care.

There is substantial body of literature from elsewhere on the effectiveness of the PMTCT programme in reducing transmission from mother to child, but data from Africa about the operational effectiveness of Option B+ in the PMTCT are sparse (17, 19–27). Particularly in Zambia the effectiveness of option B+ has not been evaluated, as far as we are aware, thereby raising concerns on its effectiveness in the elimination of MTCT. This study measured the MTCT rate of HIV on Option B+ compared to other options in cohorts of mother-baby pairs that were part of the national PMTCT programme.

## Methods

### SmartCare Design

SmartCare is an electronic health management record system which stores individual patient information at about 600 government health facilities in Zambia. It can be used for monitoring of patient treatment and outcomes and for reporting health service delivery at district, provincial and national levels across all districts of Zambia (14). SmartCare is a public domain, data system using microchip, touch screen, and solar technologies to improve health records of patient care and to enable public health reporting for persons attending health facilities. It records all patient interactions and subsequent visits to the health facility which includes clinical appointments, laboratory and pharmacy data with a unique identification number. This electronic health record system stores patient information on a computer, as well as a smart card, and easily produces reports at facility, district, provincial or national levels. SmartCare provide greater continuity of clinic based care; and increases the privacy of sensitive medical information in services for Family Planning, sexually transmitted infections and HIV. For pregnant women, SmartCare is used for Ante-natal clinical visits and enrolment into PMTCT services. SmartCare aims to reduce the burden of paperwork on health staff and improve the quality of information and decision support for patients, while providing automated information flow into the government's existing Health Management Information System (ZHMIS) (28).

### Option B+ Effectiveness Design

#### Sampling and Setting

This was a retrospective cohort study of HIV-infected women and their infants with data recorded in SmartCare. The Zambia ART programme has opt-out HIV testing for all eligible pregnant women, with all women having a positive HIV test result and those with known positive status be enrolled into the PMTCT programme. In the PMTCT programme they receive a comprehensive intervention to prevent MTCT of HIV. Since 2013, all women enrolled into the PMTCT programme are treated with lifelong ART (option B+) regardless of their CD4 cell count or WHO staging (Zambia Consolidated PMTCT Guidelines) (see Table 1).

All women enrolled in the PMTCT programme with records in the SmartCare database between 2007 and 2017 were included in the study. The outcome was measured in HEI born to women who were HIV positive.

### Data Extraction and Management

Data were abstracted from HIV infected pregnant and breastfeeding women who were enrolled into PMTCT and ART registers using SmartCare database between 2007 and 2017. All records of HEI were paired with the HIV-infected women. The extracted data included the demographic characteristics of the pregnant women at enrolment, their entry point through HIV counseling and testing, history of antenatal care for the most recent birth, full birth history on labor and delivery, ART regimen type, mode of delivery, postnatal, and follow up data for both mother and new-born baby; social-economic status; and educational attainment.

### Statistical Analysis

All analyses were done using Stata software version 14 (Stata corporation College Station, Texas). Descriptive analysis were used for the characteristics of the pregnant women and their babies. The outcome of interest was time to an HIV positive result in the babies, with those who tested HIV negative censored at the date of the negative test.

Using Kaplan-Meier (K-M), graphs were used to show time to HIV positivity, comparing women with different characteristics. Survival analysis were done to determine and compare the rate of transmission between HIV positive pregnant women on Option B+ compared with those on the other regimen in the PMTCT program. The Wilcoxon (Breslow) test was used to establish statistical significance of the difference in the survival rates between option B+ and other interventions. Both single and multiple Cox proportional hazards regression models were conducted to determine the rate of transmission of HIV and potential confounders of MTCT. The Nelson Aalen cumulative hazard estimates were used to assess the risk of transmission between the two regimen. The validity of the proportional hazard assumptions were assessed using stph-plots for the treatment regimens. Bivariate analysis using Pearson's chi squared test was used to determine the crude associations between Option B+ and infant HIV status. The rate of vertical transmission was used as a proxy to measure effectiveness at a rate of 5% or less according to WHO universal goal (4).

### Ethics Consideration

Permission was sought from Zambia Ministry of Health (MOH) and Centers for Disease Control Zambia (CDC) to use SmartCare patient data. A waiver was obtained from the University of Zambia Biomedical Research Ethics Committee (UNZABREC) reference Number 010-04-18 which granted permission to conduct this study on HIV cascade in PMTCT and associated factors. All SmartCare data had personal identifiers removed to maintain confidentiality and anonymity of the participants.

## Results

### Participation and Distribution

A total of 1,444 mother and their infants were matched to the SmartCare record for their infants and included in the analysis. The mothers were aged 15–50 years with a Median (IQR) age of 33 (28–38) years. Further, 87% (1,185) were married and 56% (660) had attained primary education only. In addition, 50% (710) of the mothers reported a parity of (0–1) whilst 10% (145) had five or more previous births, but normal vaginal delivery was reported by almost all 97% (1,386) women.

The mean baseline CD4^+^ cell count was 467 cells/ml (SD 246.5). Of the total women, 40% (580) had been enrolled on Option B+, of which only 1.4% (12) reported non-adherent (Table 2).

**Table 2:** Characteristics of HIV positive women on Lifelong ART (Option B+) from Zambia SmartCare routinely collected data, 2005–2017.

**Characteristic**	**Frequency**	**(%)**	**95% CI**
**Age categories (in years)**			
15–24	123	8.5	[7.2–10.1]
25–34	697	48.3	[45.7–50.9]
35–50	624	43.2	[40.7–45.8]
**Marital status**			
Single	136	9.9	[8.5–11.6]
Married	1,185	86.6	[84.7–88.3]
Divorced/Widowed	47	3.5	[2.6–4.5]
**Education level**			
None/Primary	660	56.2	[53.3–58.9]
Secondary	472	40.2	[37.4–43.0]
Tertiary	43	3.6	[2.6–4.5]
**Parity**			
0–1	710	49.2	[46.6–51.8]
2–4	588	40.8	[38.2–43.3]
5–8	145	10.0	[8.6–11.7]
**Delivery mode**			
Assisted	25	1.8	[1.2–2.6]
Cesarean	18	1.3	[1.0–1.9]
Normal delivery	1,386	96.9	[95.9–97.8]
**Current CD4 count**			
11–349	330	35.0	[32.0–38.1]
350–499	255	27.1	[24.3–30.0]
500–1550	357	37.9	[34.8–41.0]
**Had trouble taking pills**			
No	1,008	98.9	[98.1–99.4]
Yes	11	1.1	[0.6–1.9]
**Number of doses missed**			
0	815	98.6	[97.5–99.2]
1 or more	12	1.4	[0.8–2.5]

*Sample size = 1,444*.

*Median (IQR) age = 33 (28–38) years*.

*Mean baseline CD4^+^ cell count = 467 cells/ml (SD 246.5)*.

*95% CI obtained using Pearson chi squared*.

HEI of positive mothers on option B+ regimen had a reduced transmission rate of 2.9% (17/580) compared to other regimen (*P* = 0.003). MTCT was higher among the women aged 25–34, accounting for 7% (46/697) (*P* = 0.034). Furthermore, married women had a higher transmission rate of 12% (6/47) compared to those not married 5% (60/1,125) (*P* < 0.001). HIV positive mothers who delivered through assisted means had a higher likelihood to transmit the Virus to their infant, 12% (3/25) compared to those with a spontaneous vaginal delivery 5% (70/1,386) or cesarean section 0% (0/18) (*P* = 0.184) (Table 3).

**Table 3:** Bivariate analysis of background characteristics and Regimen type of HIV Positive mothers from Zambia SmartCare routinely collected data, 2007–2017.

		**Infant HIV status**				
***N* = 1,444**		**Negative**		**Positive**		
**Characteristic**	**Total**	**Frequency**	**(%)**	**Frequency**	**(%)**	***P*-Value**
**Treatment group**						0.003^*^
Option A/B	864	808	[93.5]	56	[6.5]	
Option B+	580	563	[97.1]	17	[2.9]	
**Age categories (in years)**						0.034^*^
15–24	123	118	[95.9]	5	[4.1]	
25–34	697	651	[94.4]	46	[6.6]	
35–50	624	602	[96.5]	22	[3.5]	
**Marital status**						<0.001^**^
Single	136	136	[100.0]	0	[0.0]	
Married	1,185	1,125	[94.9]	60	[5.1]	
Divorced/Widowed	47	41	[87.2]	6	[12.8]	
**Education level**						0.336^**^
None/Primary	660	629	[95.3]	31	[4.7]	
Secondary	472	446	[94.5]	26	[5.5]	
Tertiary	43	39	[90.7]	4	[9.3]	
**Parity**						0.302^**^
0–1	710	669	[94.2]	41	[5.8]	
2–4	588	560	[95.2]	28	[4.8]	
5–8	145	141	[97.2]	4	[2.8]	
**Delivery mode**						0.184^**^
Normal Delivery	1,386	1,316	[95.0]	70	[5.0]	
Assisted	25	22	[88.0]	3	[12.0]	
Cesarean	18	18	[100.0]	0	[0.0]	
**Current CD4 count**						0.465^*^
11–349	330	309	[93.6]	21	[6.4]	
350–499	255	232	[91.0]	23	[9.0]	
500–1550	357	328	[91.9]	29	[8.1]	
**Had trouble taking pills**						0.548^**^
No	1,008	954	[94.6]	54	[5.4]	
Yes	11	11	[100.0]	0	[0.0]	
**Number of doses missed**						0.525^**^
0	815	772	[94.7]	43	[5.3]	
1 or more	12	12	[100.0]	0	[0.0]	

*Sample size = 1,444*.

*Median (IQR) age = 33 (28–38) years*.

*Mean baseline CD4^+^ cell count = 467 cells/ml (SD 246.5)*.

* *P-Values obtained using Pearson chi squared*.

** *P-Values obtained using Fishers Exact*.

*Bold values represent a significance of p < 0.2*.

### Key Predictors for Mother to Child Transmission of HEI

In the survival analysis, overall, a total follow up time of 38,520 months was experienced by 1,444 children born to HIV positive mothers. There were 73 recorded HIV positive tests, giving a MTCT of 5.1 per 100 livebirths. A Kaplan-Meier (K-M) estimate showed that HIV exposed infants of mothers recruited on option B+ had lower MTCT than those recruited on the other options. A Wilcoxon (Breslow) test for equality of survival functions showed statistical significance (chi2 = 4.95, *P* = 0.025) for the observed difference in HIV survival rates between Option B+ and other PMTCT options. Proportional hazard assumptions were assessed using stph-plots for treatment regimen and were satisfied.

**Figure 1:**
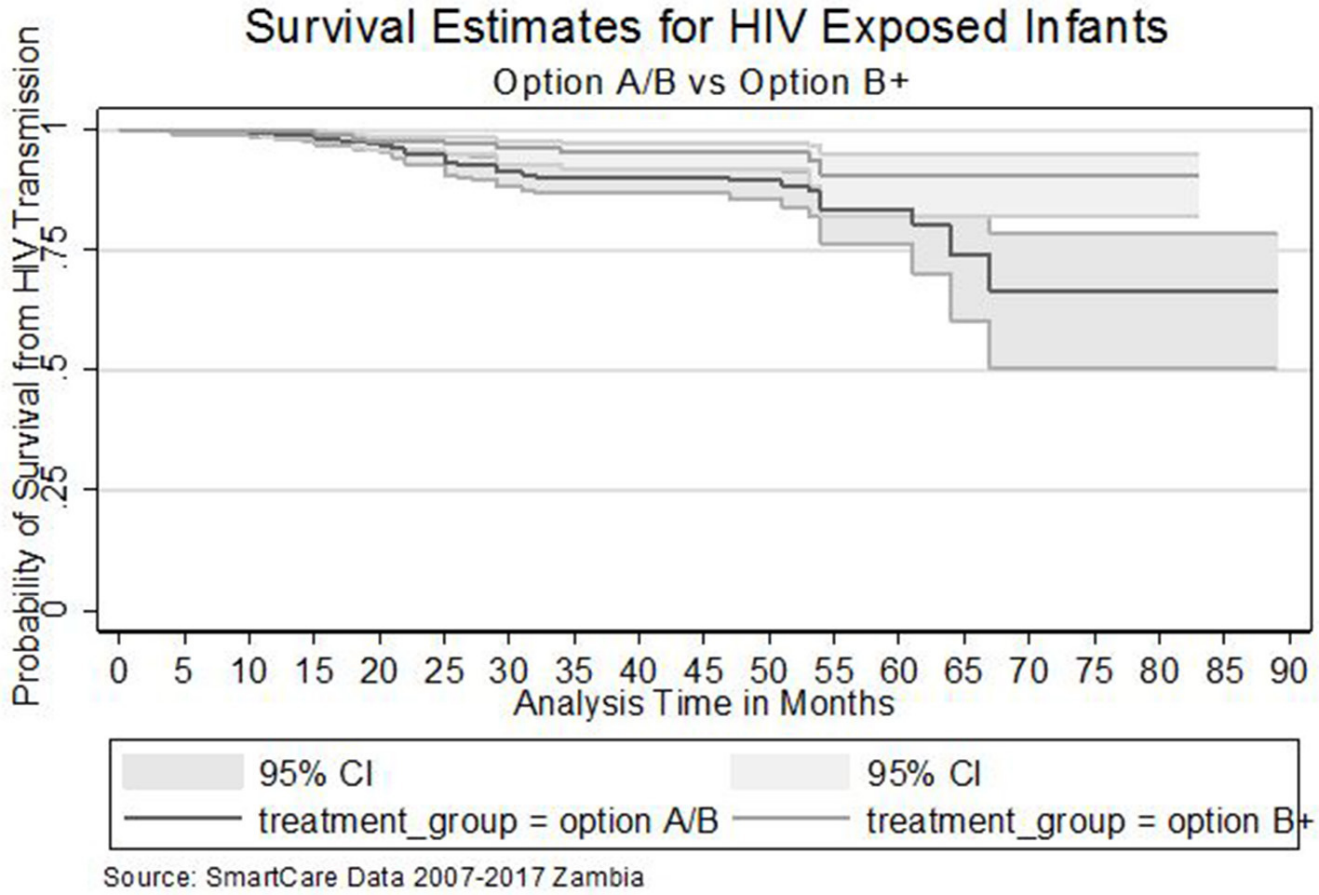
Comparison of survival estimates between exposed infants of HIV positive mothers on option A/B and Option B+. (a) Zambia introduced lifelong Option B+ for HIV positive mothers mid 2013. (b) Prior to 2013, HIV positive mothers with CD4 count <350 cells/ ml were put on Option A/B.

**Figure 2:**
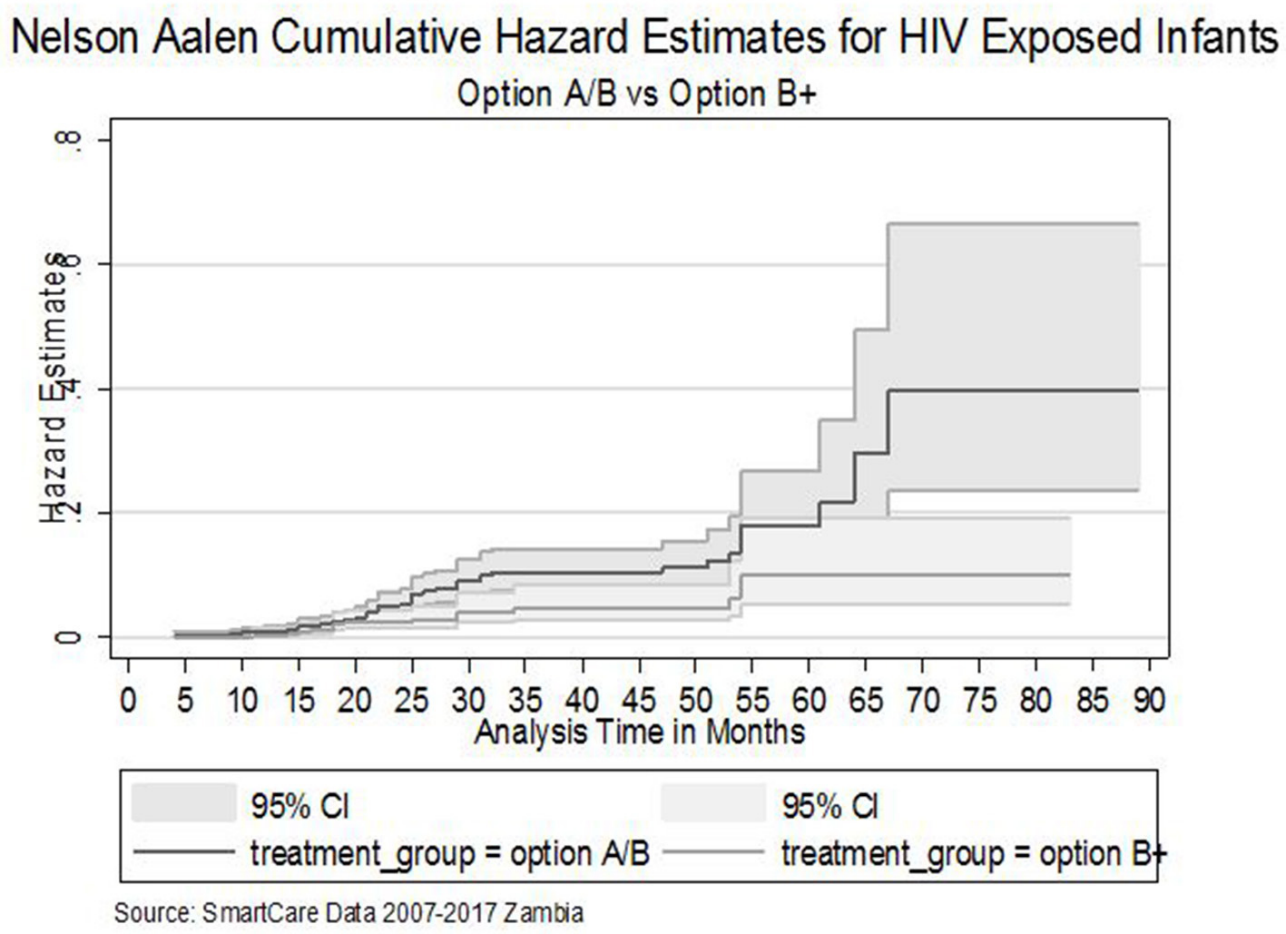
The Nelson Aalen cumulative hazard estimates for HIV exposed infants by treatment groups.

**Figure 3:**
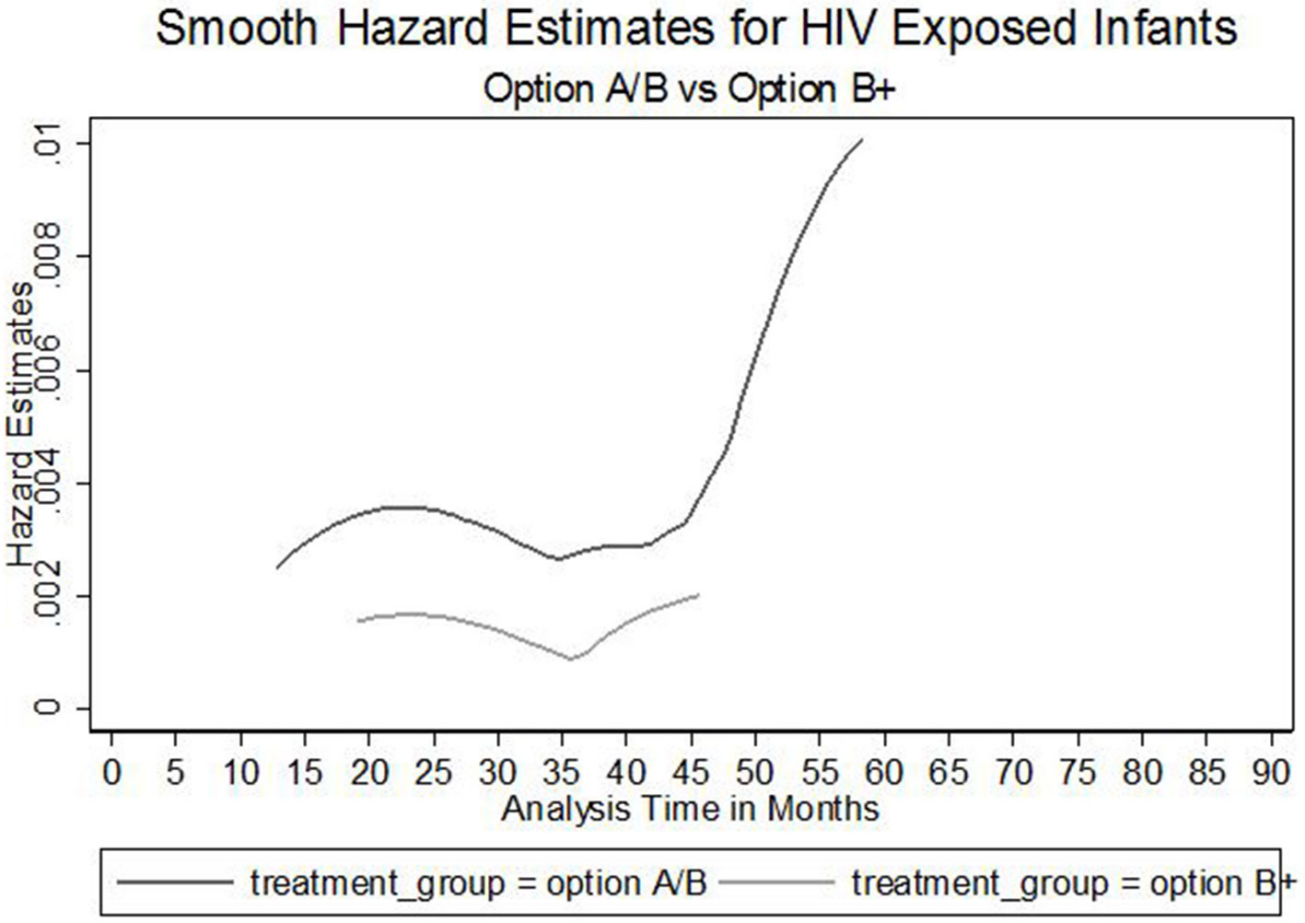
Smooth hazard estimates for HIV exposed infants by treatment groups.

Furthermore, The Nelson Aalen cumulative hazard estimates (HR = 0.63, *P* = 0.025) and the smooth hazard estimates indicated similar evidence of statistical significance for a difference in transmission of HIV between infants exposed to Option B+ and Option A/B mothers (Figures 1–3).

Exposed infants to HIV positive option B+ mothers had 50% reduced risk of having HIV infection through vertical transmission compared to those exposed to option A/B mothers [adjusted HR = 0.4; 95% CI = 0.28–0.84; *P* = 0.010]. HEI to option B+ women who were married had an increased risk 50% of getting infected compared to those exposed to mothers not married [adjusted HR = 1.5; 95% CI = 3.43–6.30; *P* < 0.001]. Furthermore, Exposed infants whose mothers had assisted delivery had 3 times increased risk of getting infected compared to those who had normal vaginal delivery [Adjusted HR = 3.2; 95% CI = 0.98–10.21; *P* = 0.050] (Table 4).

**Table 4:** Cox proportional hazard analysis of background characteristics and Regimen type of HIV Positive woman from Zambia SmartCare routinely collected data, 2018.

**Characteristic**	**Unadjusted-HR**	***P*-Value**	**95% CI**	**Adjusted-HR**	***P*-Value**	**95% CI**
**Treatment group**						
Option A/B	Ref.			Ref.		
Option B+	0.4	0.004		0.5	0.010	[0.28–0.84]
**Age group (in years)**						
15–24	Ref.					
25–34	1.3	0.531	[0.53–3.39]			
35–50	0.6	0.359	[0.24–1.68]			
**Education level**						
None/Primary	Ref.					
Secondary	1.3	0.391	[0.75–2.12]			
Tertiary	1.4	0.489	[.51–4.10]			
**Parity**						
0–1	Ref.					
2–4	0.9	0.527	[0.53–1.39]			
5–8	0.4	0.087	[0.15–1.38]			
**Marital status**						
Single	Ref.			Ref.		
Married	1.5	<0.001	[3.60–6.65]	1.5	<0.001	[3.43–6.30]
**Current CD4 cell count**						
11–349	Ref.					
350–499	1.4	0.256	[0.78–2.55]			
500–1550	1.2	0.485	[0.69–214]			
**Delivery mode**						
Normal vaginal delivery	Ref.			Ref.		
Assisted delivery	2.1	0.215	[0.65–6.69]	3.2	0.050	[0.98–10.21]
Cesarean	1.7	1.000	[a]	4.0	1.000	[a]

*[a] Missing Confidence intervals because of missing standard errors due to stratum with single sampling unit*.

## Discussion

In 2014, the national HIV vertical transmission rate recorded was at 9% (29). Our current data suggest an HIV transmission rate of 5%. This finding is actually lower than sub-Saharan average rate as observed in one cohort study conducted in Ethiopia and other African countries which showed that out of the 221 live births from HIV positive mothers, MTCT rate was approximately between 8 and 10% (30). The Global eMTCT Plan recommends providing comprehensive PMTCT services to at least 95% of pregnant women and reduce MTCT to <5% by the year 2015 and zero transmission by 2030 (31).

The level of HIV testing uptake among ANC women has substantially increased from the time PMTCT was introduced in Zambia in 1999. However, failure to attend all clinical appointments, adherence to treatment for option B+ mothers contributes to missed opportunities for early infant Diagnosis (EID), and as a result many are not tested until after 24 months as observed in this study. Similar findings were observed in a retrospective follow up study in Sub-Saharan Africa, from 2004 to 2009 on HIV testing of infants ≥18 month, which posed a challenge as only 896 (10.6%) of infants completed the follow up HIV testing, of which 106 infants were found to be positive representing 14.3% vertical transmission rate (30). Among the key challenges faced in the diagnosis of infants are lack of training to collect and handle Dry Blood Sample (DBS), results not collected from central laboratory and misplacement of results within the health facility before reaching the mothers. An infant is presumed HIV uninfected if they had negative DNA PCR assays at 6 and 14 weeks of age. A child is classified as HIV uninfected if both antibody tests are negative at or after 18 months. Its only by addressing these challenges that option B+ benefits can be realized.

Several potential limitations to this study were observed. Firstly, the main limitation was the inability of SmartCare to link mothers to their infants, which was very crucial for this study as it affected the sample size. Secondly, Option B+ (lifelong ART) coverage in Zambia was gradual after its adoption in 2013 and many of the health facilities were still using option A/B and this could have reduced the effect of change. Besides data was available only in facilities that had SmartCare system active and functional. Another limitation is that being routinely collected data, critical variables such as EID, mother-baby pair link, retention patterns, lost to follow up, and heath care utilization including the frequency of a patient's appearance in the ANC records were missing. The frequency of a patient's health care utilization could have been used to adjust for those visiting the heath care facility more often which would have indicated who experienced the event more quickly, and thus bias the time-to-event analysis. Despite these challenges, however, the data provides better estimates on the effectiveness of option B+. Besides, most of the facilities affected with option B+ roll-out were mainly in rural areas. Furthermore, considering that this is one of the first studies, as we are aware, to document the experience of implementing option B+ in Zambia which will help accelerate toward a 2030 ambitious goal of zero HIV transmission, we believe this study is very worthy to be undertaken.

Option B+ regimen for mothers and infants offers significant benefits for transmission prevention, maternal health and public health program delivery. It presents distinct advantages in terms of transmission prevention to uninfected partners and increased simplicity potentially improving program feasibility, access, uptake and cost effectiveness. Despite these benefits, however, concerns have been raised about the safety of ART exposure to fetuses and infants as well as adherence challenges for pregnant and breastfeeding mothers (32). Similar Option B+ benefits were observed in a cohort comparative study of 102 women on ART prior to Option B+ to a cohort of 109 women on Option B+ conducted in Malawi, which showed that women on Option B+ had fewer WHO 3/4 conditions, higher CD4 count and lower mortality compared to those in pre Option B+ (22). This high mortality and poor health of pre option B+ women posed a direct effect on the health and survival of their infants. This is because pregnant women with a high viral load and lower CD4 count are more likely to transmit HIV virus to their new-born babies (33). Furthermore, in another study, women on option B+ had low mortality compared to those on CD4 cell count or WHO clinical stage criterion group. Mortality among the women on option B+ during pregnancy was 0.4% while those enrolled based on CD4 cell count or WHO clinical stage 3/4 criterion recorded mortality of 3% (17).

Another study conducted on retention of pregnant and breastfeeding women in Malawi also observed that most women (83%) starting ART with Option B+, over 17% were lost to follow up in the 6 months period and most of them occurred in the first 3 months of therapy. The results further showed that option B+ women who started therapy during pregnancy were 5 times more likely than pre option B+ women never to return for their next clinical follow up (34). These results indicate that although Option B+ therapy possibly had a better outcome, retention of women in care and lost to follow up especially after delivery was a challenge. The possible explanation could have been that women started on option B+ still enjoyed a good measure of health because of their good immune system, having a high CD4 cell count and low viral copies, and would not follow up their clinical appointments and adhere to therapy potentially increasing the chances of HIV transmission to the infant (22, 33). Universal access to HIV testing in ANC and 100% linkage to care and treatment coupled with strategies to improve retention and adherence to treatment is crucial to further reduce vertical transmission rate.

## Conclusion

In Zambia Option B+ has been found to be more effective in reducing MTCT rates to lower acceptable levels than any other options thus opening opportunities for scaling up access to life-long ART and improving retention and contribute to potentially reduced vertical transmission sustainably. However, these findings also suggest the need for programmatic efforts to identify other maternal health survival bottlenecks that could hamper universal access to PMTCT interventions for all mother-baby pairs on lifelong ART in poorly accessed groups. This may include strategies to prevent missing of clinical appointments, infant post-natal follow up and eventual non-retention. Lastly but not the least, these findings also indirectly suggest the need for further integration of ANC services to include innovative PMTCT interventions as part of a total service package.

## Recommendations

Since Early Infant Diagnosis at recommended time is directly linked to care and treatment, supporting existing Government measures to retain HIV-infected women in eMTCT programme in order to improve access to universal HIV treatment and care among women is key in addressing barriers to increased uptake of PMTCT. Strengthening HIV testing in ANC especially in rural health facilities, encouraging women to adhere to treatment and attend all clinical appointments as well as providing initiatives that seek to overcome barriers to treatment are some of the ways that can help improve maternal and new-born health. Furthermore, health workers should ensure that HIV-infected women, on option B+ are retained in care and bring their babies for clinical appointments and testing at recommended schedules.

## Data Availability Statement

The data that support the findings of this study are available from the Zambia Ministry of Health. These datasets can be obtained on request from the Zambia Ministry of Health.

## Ethics Statement

Permission was sought from Zambia Ministry of Health (MOH) and Centers for Disease Control Zambia (CDC) to use SmartCare patient data. A waiver was obtained from the University of Zambia Biomedical Research Ethics Committee (UNZABREC) reference Number 010-04-18 which granted permission to conduct this study on HIV cascade in PMTCT and associated factors. All Smartcare data had personal identifiers removed to maintain confidentiality and anonymity of the participants.

## Author Contributions

BM conceived the study ideas, design, analyzed data, wrote the draft manuscript, and wrote the final manuscript. CM participated in the study design, methods, analysis, and edited the final manuscript. PMe contributed to the analysis, edited the manuscript, and made contributions to the final analysis. JT participated in the study design, methods, analysis, edited the manuscript, and contributed to the final analysis. PMu edited the manuscript and contributed to the final analysis.

### Conflict of Interest

The authors declare that the research was conducted in the absence of any commercial or financial relationships that could be construed as a potential conflict of interest.

## Abbreviations

MTCT: Mother to Child Transmission
PMTCT: Prevention of mother to Child Transmission
EID: Early Infant Diagnosis
HEI: HIV Exposed Infants
ANC: Antenatal Care
eMTCT: elimination of Mother to Child Transmission
ART: Antiretroviral Therapy
cART: Combined Antiretroviral Therapy
HIV: Human Immuno-Virus
DBS: Dry Blood Sample
DNA PCR: Deoxyribonucleic Acid Polymerase Chain Reaction
CDC: Centers for Disease Control and Prevention
UNZABREC: University of Zambia Biomedical Research Ethics Committee
MOH: Ministry of Health
WHO: World Health Organization.
